# Comparing the Therapeutic Effects of Garlic Tablet and Oral Metronidazole on Bacterial Vaginosis: A Randomized Controlled Clinical Trial

**DOI:** 10.5812/ircmj.19118

**Published:** 2014-07-05

**Authors:** Farnaz Mohammadzadeh, Mahrokh Dolatian, Masoome Jorjani, Hamid Alavi Majd, Nasrin Borumandnia

**Affiliations:** 1 Department of Midwifery, International Branch, Shahid Beheshti University of Medical Sciences, Tehran, IR Iran; 2 Department of Midwifery and Reproductive Health, Shahid Beheshti University of Medical Sciences, Tehran, IR Iran; 3 Department of Pharmacology, Shahid Beheshti University of Medical Sciences, Tehran, IR Iran; 4 Department of Biostatistics, Shahid Beheshti University of Medical Sciences, Tehran, IR Iran

**Keywords:** Vaginosis, Bacterial, Garlic, Metronidazole

## Abstract

**Background:**

Bacterial vaginosis (BV) is one of the most common gynecological infections during reproductive age. Although metronidazole is one of the most effective medications recommended as the first-line treatment, it has various side effects. Because of the side effects and contraindications of some chemical medicines, using herbs has been investigated in treating BV.

**Objectives:**

The aim of this study was to compare the effect of garlic tablet (Garsin) and oral metronidazole in clinical treatment of the BV in women referred to Resalat Health Center, affiliated with Mazandaran University of Medical Sciences, in 2013.

**Patients and Methods:**

This randomized clinical trial was conducted on 120 married women aged 18 to 44 years who were diagnosed with BV by Amsel’s clinical criteria and Gram staining. Enrolled women were randomly allocated to two groups of 60 patients and were treated with either garlic tablet or oral metronidazole for seven days. Amsel’s criteria and Gram stain were assessed seven to ten days after beginning the treatment period and side effects were registered.

**Results:**

Amsel’s criteria were significantly decreased after treatment with garlic or metronidazole (70% and 48.3%, respectively; P < 0.001). Therapeutic effects of garlic on BV were similar to that of metronidazole (63.3% and 48.3%, respectively; P = 0.141). There were significant differences between the two treatment groups in terms of side effects; metronidazole was associated with more complications (P = 0.032).

**Conclusions:**

This study reveals that garlic could be a suitable alternative for metronidazole in treatment of BV in those interested in herbal medicines or those affected by side effects of metronidazole.

## 1. Background

Bacterial vaginosis (BV) is one of the most common causes of vaginal discharge during reproductive age (1). The prevalence of BV varies with regard to the geographical location, socioeconomic status, and race and has been reported between 8% and 51% in different populations (2). In Iran, its prevalence was reported to be 39.9% in Brojen (3), 21.1% in Lahijan (4), 16.2% in Zanjan (5), and 28.5% in Hamadan (6). According to a report by the Center for Disease Control (CDC), the prevalence of BV in the United States was 29.2% among 14 to 49-years-old women and 25% among pregnant women (7). This infection results from reduced quantity of hydrogen peroxide producing lactobacillus and increased anaerobic organisms such as *Gardnerella vaginalis*, *Mycoplasma hominis*, and *Prevotella *species (8).

This infection is asymptomatic in 50% to 75% of cases and symptomatic cases present with homogeneous gray-white vaginal discharge with fishy odor, especially after intercourse or during menstruation (9). BV is associated with several unpleasant complications such as chorioamnionitis, preterm rupture of the membranes, premature labor, vaginal cellulite after hysterectomy, endometritis, cervicitis, cervical intraepithelial neoplasia, infertility, and recurrent pelvic pain (10). Although metronidazole is considered as the first-line of treatment according to CDC recommendations, beneficial effects of this drug should be weighed against its side effects including diarrhea, vomiting, metallic taste, headache, and dizziness. Gastrointestinal disorders are the major complications of metronidazole use that occur during treatment and may lead to discontinuation of treatment (11). Clindamycin is the next antibiotic of choice for BV treatment and is associated with such complications as abdominal cramps, difficile colitis, nausea, vomiting, diarrhea, and elevated liver enzymes (12). Statistics related to the expected rate of cure with this drug suggest its low efficacy in practice (13). Considering the strong history of medical sciences in Iran, use of herbal medicine has a special place and there has been a growing trend toward public use of herbal medicine (14). Tea leaf oil and garlic can be used as alternative herbal medications for BV (15). Garlic, with scientific name of *Allium sativum*, is from *Liliaceae* family. It is an herbaceous bulbous plant and the bulb constitutes its medicinal part (16). The healing and antibacterial properties of garlic have been known for a long time. Some beneficial effects of garlic on humans include lowering blood pressure, reducing cholesterol and serum triglycerides, and preventing atherosclerosis and cardiovascular diseases (17). The ajoene in garlic decreases blood viscosity and improves some types of anemia (18). Sulfur and selenium in garlic prevent cancer and if the cancer has already developed, reduce its size (19).

Garlic contains 33 sulfuric compounds; allein is responsible for its antibacterial properties. When garlic is crushed, the odorless allein compound touches alleinase and turns into allicin, which is an odorous substance (20). Allicin works through disrupting oxidation of thiol group of the bacterial enzymes and disruption in synthesis of bacterial RNA, proteins, and enzymes (21). Clinical evidence have revealed that oral preparations of garlic can be as effective as metronidazole in treatment of *Trichomonas vaginalis* (22). Moreover, vaginal cream made of garlic has had satisfactory effects in treatment of vaginal candidiasis (23). Garlic vaginal cream seems to be an appropriate alternative to metronidazole vaginal gel in treatment of BV. Patient complaints and Amsel’s clinical criteria showed a significant decrease following treatment with garlic vaginal cream and metronidazole vaginal gel (24). Alliums (garlic, onion, and leek) contain antimicrobial components that affect the microbes associated with spontaneous preterm delivery and decreased risk of preterm labor (25). Although clinical studies have shown women’s difficulties with use of vaginal products (26), sufficient studies have not been conducted regarding the therapeutic effects of garlic tablets on BV. Considering complications associated with chemical drugs, the need for an alternative treatment with fewer complications, and tendency of many people toward herbal medicines, this study was conducted to compare the efficacy of garlic tablet and metronidazole in treatment of BV.

## 2. Objectives

Given the high prevalence of BV and its negative effects on sexual and individual life, this study aimed to compare the effect of garlic tablet and metronidazole in treatment of BV in women referred to Resalat Health Center, affiliated with Mazandaran University of Medical Sciences, in 2013.

## 3. Patients and Methods

To compare the therapeutic effects of garlic tablet and oral metronidazole on BV in this single-blind randomized controlled clinical trial, 120 women referred to the Resalat Health Center of Amol Town (public referral center) between July 2013 and January 2014, were recruited.

Married women aged between 18 and 44 years were recruited. Those with the following conditions were excluded from the study: pregnant or lactating women; injection-drug users; taking immunosuppressive medicine, vaginal drugs, or antibiotics during the preceding two weeks; participating in other research trials during the preceding four weeks; and being diagnosed with other diseases such as thyroid disorders, diabetes, or cervicitis. The participants had no premature menopause, mental disorder, vaginal candidiasis, or *T. vaginalis* infection (based on direct smears). Moreover, the criteria for recruiting participants were based on the presence of at least three out of the four Amsel’s criteria including presence of clue cells in the vaginal fluid, presence of fishy odor, thin grey-white homogenous discharge, and vaginal pH of more than 4.5. Gram stain diagnosis of BV was made by score larger than three by Nugent criteria. Patients would be dropped out of study if they experienced any of the following condition during treatment: the urge to use antibiotics, intolerance to any of the study medications, discontinuation of medications for at least 48 hours, and dietary garlic consumption during treatment. Study was approved by the Ethics Committee of the International Division of Shahid Beheshti University of Medical Sciences and Health Services (Ethical code: 116/2880; date: 8/10/2013) and was registered in Iranian Registry of Clinical Trials (Registration ID: IRCT201207153226N4). For sample size calculation, we used findings from similar studies in comparable populations (24). Therefore, the number of participants required to detect a standardized difference in treatment response between groups was calculated at 120 (60 per group) with 80% power using a cutoff score for statistical significance of 0.20 and with considering up to 15% lost to follow-up (27):

$$n=\frac{{(z_{1-\alpha /2}+Z_{\beta })}^{2}}{\varepsilon^{2}}[\frac{P_{1}(1-P_{1})}{k}+P_{2}(1-P_{2})]$$

P _1_ = 0.70

P _2_ = 0.90

ε = P _1_-P _2_ = 0.20

α = 0.05 (⇒ Z _(1-α/2)_ = 1.96)

1-β = 0.80 (⇒ Z _(1-β)_ = 0.84)

K = 1

Our study population were women aged 18 to 44 years, married, with vaginal discharge who were referred to Resalat Health Center. The interested women were invited to participate in this study by furnishing them with information, the goals of the study were explained to them, and all of them signed an informed consent. In order to enroll the participants, they were given a questionnaire consisted of individual and demographic, menstrual, pregnancy, medical history, and health information. After taking history and vaginal examination by speculum with the patient in lithotomy position, a sterile cotton swab was used to collect discharge specimens from lateral vaginal walls and posterior fornix and was immediately placed on three smear slides. The first slide was examined for *T. vaginalis *and clue cells under a microscope after adding one to two drops of normal saline. After adding a drop of 10% KOH, the second slide was examined by Whiff test and assess by microscope to detect *Candida* *albicans *mycelium and hyphae. After air drying, the third smear sample was sent to the laboratory where it was examined after Gram staining by a microbiologist (blind to clinical results) under the light microscope with magnification of × 1000 and immersion oil.

Nugent criterion was used for interpretation of smear slides and diagnosis of BV using Gram stain. Test results were ready within 24 to 48 hours. In this method, a rating system of zero to ten was used and scores above three were considered as diagnostic for BV. Samples were excluded from the study when infections with *C. albicans* or *T. vaginalis *were diagnosed. Criteria for diagnosis of BV included presence of three out of four Amsel’s clinical criteria including pH > 4.5, positive Whiff test results, a gray-white homogenous discharge, and presence of clue cells and Nugent score of four to ten in Gram staining.

Among women complaining of vaginal discharge who were referred to the Resalat Health Center of Amol Town, affiliated with Mazandaran University of Medical Sciences, 360 women volunteered to participate in the study and filled the questionnaire. Amongst the volunteers, 220 were excluded due to lack of inclusion criteria and were referred to gynecologists for treatment. A total of 140 women were recruited with convenience sampling method (flowchart of participants) according to the eligibility criteria. The random allocation was achieved by www.random.org with blocking method (size of blocks: 4 and 6). Therefore, samples were coded and allocated randomly to two equal groups of 70 participants treated with garlic tablet and oral metronidazole. The researcher put the drugs of each code in an envelope with the respective code written on the envelope. The garlic tablets were produced in the same shape, color, and odor as metronidazole tablets.

To assess the validity of the questionnaire and checklists, we used qualitative content analysis. Questionnaires and checklists were sent to ten specialists in the field of the study and the material was revised and improved according to the received feedbacks. The microscope was purchased from Leica Co., the Unite States. The magnification for each mode was calibrated using a standard microscope in the laboratory. Vaginal discharge pH was measured by paper pH-meter bands (Nagel-Germany-Machery Co) and calibrated with the standard paper pH-meter bands in the laboratory. In order to achieve observations validity, a researcher was trained by a microbiologist and the diagnosis was confirmed by the same microbiologist. To determine observations reliability, Kappa statistics were used for measuring agreement. Ten participants were questioned and examined simultaneously by a researcher and a colleague in the research environment. Forms were completed and Kappa coefficient was calculated. Kappa coefficient was at least 0.8; therefore, questionnaire and checklist reliability was approved. To determine pH-meter paper reliability, five samples were prepared by a research unit and pH was measured; with the same results, reliability was confirmed. To determine reliability of laboratory results, stained coded slides were read in the laboratory by a skilled microbiologist. Then the code of the slides number ten through 15 were changed and presented to the same microbiologist; Kappa coefficient showed 0.8; thus, the reliability was approved. To determine the reliability of the researcher's observations, wet-sample slides numbered ten to 15 were read by a microbiologist and a researcher simultaneously. Kappa coefficient showed 0.8; hence, the reliability was approved.

The drugs consisted of garlic in 500-mg tablets that contain garlic powder (85.42%; equivalent to 8.9-mg allein) (Goldaroo Pharmaceutical Co., Isfahan, Iran, registration No. 1228030383), and 250-mg metronidazole tablets (Tehran Chimi Co., Iran, registration No. 1228035791). Both drugs were taken with meal at the dose of two tablets each 12 hours for seven days. To ensure correct use of medication and record information, participants were issued with self-report forms to register daily complaints and record drug use. Participants were also trained on how to fill out the forms. Participants were reminded that if they had problems, they should either attend the center or contact the researchers. To ensure correct use of medications, the researcher remained in touch with study subjects via telephone. Amsel’s and Nugent criteria were once again assessed seven to ten days after commencement of the treatment course. Presence of one or none of the Amsel’s criteria and Nugent score of zero to three meant successful treatment. 

Data were analyzed with SPSS16 software (SPSS Inc., Chicago, IL, USA) using independent-samples and paired-samples t test, GEE logistic regression, Chi square, Fisher’s exact test, and Mann-Whitney U test.

## 4. Results

In this study, 20 patients were excluded: nine due to lack of timely referral; four due to becoming pregnant during the study; one due to bleeding during the study; three because of intolerance to metronidazole; one because of intolerance to garlic; and two because of taking antibiotics during the study. 

A total of 120 women, 60 in each group, completed the study. There was no significant difference between two groups in terms of demographic and reproductive characteristics such as age, duration of marriage, age at first pregnancy (t-test; P > 0.05), parity, abortion, and frequency of intercourse per week (Mann-Whitney U test; P > 0.05). The two groups were similar in terms of contraceptive method and history of vaginitis (Chi square; P > 0.05) (Table 1).

Amsel's criteria were significantly decreased after treatment with garlic or metronidazole, but the difference between two groups in terms of clinical improvement, i.e. improvement in Amsel’s criteria, was significant; in other words, garlic was more effective than metronidazole in terms of clinical improvement (Table 2). The results of mixed model analysis or GEE analysis are shown in Table 3; the effect of treatment group on Amsel’s criteria was significant (P > 0.05). In terms of improvement in laboratory results, i.e. improvement in Nugent criteria, the difference between two groups was not significant and both drugs had similar effects on improving Nugent criteria (Table 4). There were no significant differences between two groups in terms of treatment success rate, i.e. concurrent improvement in Amsel’s and Nugent criteria (Table 5). Nausea was reported in 5% of subjects in garlic group while none of the subjects in metronidazole group showed this symptom. Heartburn had the highest frequency among study subjects in garlic (10%) and metronidazole (15%) groups. Vomiting, diarrhea, headache, metallic taste, or skin rash were not reported in any of the subjects in garlic group while these complications were respectively 1.7%, 1.7%, 6.7%, 11.7%, and 1.7% in metronidazole group. This study revealed significant differences between garlic and metronidazole groups regarding side effects. In other words, treatment with metronidazole was associated with more side effects (Table 6).

**Table 1.** Demographic Characteristics and Confounding Factor of the Participants ^a^

Group	Garlic (n = 60)	Metronidazole (n = 60)	P Value
**Age, y**	29.58 ± 6.30	30.35 ± 5.58	0.481
**Duration of Marriage, y**	11.08 ± 6.66	11.42 ± 6.32	0.779
**Age at the First Pregnancy, y**	19.42 ± 3.18	20.34 ± 3.35	0.409
**Parity**	1.83 ± 1.81	1.70 ± 1.15	0.378
**Abortion**	0.57 ± 0.72	0.47 ± 0.77	0.227
**Intercourse Per Week**	1.65 ± 0.90	1.83 ± 0.87	0.182
**Contraception**	-	-	< 0.999
**History of Vaginitis**	-	-	0.670

^a^ Data are presented as Mean ± SD.

**Table 2.** Comparison of Clinical Improvement Regarding Amsel’s Criteria in Women With Bacterial Vaginosis ^a,b^

Group	Garlic	Metronidazole	Total
**Clinical Improvement**	42 (70)	29 (48.3)	71 (59.2)
**Lack of Clinical Improvement**	18 (30)	31 (51.7)	49 (40.8)
**Total**	60 (100)	60 (100)	120 (100)

^a^ Data are presented as No. (%).

^b^ Chi square = 5.829 and P > 0.025.

**Table 3.** GEE Model of Gray-White Homogenous Discharge, Positive Whiff Test Results, Clue Cell, and pH > 4.5

	Estimate	Standard Error	P Value
Gray-White Homogenous Discharge			
Metronidazole	B = 0.810	0.3551	0.023
Garlic		Reference group	
**Positive Whiff Test**			
Metronidazole	B = 0.246	0.3416	0.472
Garlic		Reference group	
**Clue Cell**			
Metronidazole	B = 0.129	0.3514	0.715
Garlic		Reference group	
**pH > 4.5**			
Metronidazole	B = -1.936	0.9944	0.051
Garlic		Reference group	

**Table 4.** Comparison of Laboratory Improvement in Women With Bacterial Vaginosis ^a,b^

Group	Garlic	Metronidazole	Total
**Lab Improvement**	41 (68.3)	33 (55)	74 (61.7)
**Lack of Lab Improvement**	19 (31.7)	27 (45)	46 (38.3)
**Total**	60 (100)	60 (100)	120 (100)

^a^ Data are presented as No. (%).

^b^ Chi square = 2.256 and P > 0.05.

**Table 5.** Comparison of Treatment Success in Women With Bacterial Vaginosis ^a,b^

Group	Garlic	Metronidazole	Total
**Successful Treatment**	38 (63.3)	29 (48.3)	67 (55.8)
**Failure in Treatment**	22 (35.7)	31 (51.7)	53 (44.2)
**Total**	60 (100)	60 (100)	120 (100)

^a^ Data are presented as No. (%).

^b^ Chi square = 2.737 and P > 0.05.

**Table 6.** Comparison of Medication Side Effects in Women With Bacterial Vaginosis ^a,b^

Group	Garlic	Metronidazole	Total
**With Side Effect**	9 (15)	20 (33.3)	29 (24.2)
**Without Side Effect**	51 (85)	40 (66.7)	91 (75.8)
**Total**	60 (100)	60 (100)	120 (100)

^a^ Data are presented as No. (%).

^b^ Chi square = 5.502 and P > 0.032.

## 5. Discussion

This was the first study that showed garlic tablet and oral metronidazole had similar effects in treatment of BV. In vitro studies and evaluation of results of antibacterial activity of alcoholic garlic extract have shown its significant effect on gram-positive bacteria (28). In a study, the significant antibacterial effect of pure garlic extract on gram-positive and gram-negative bacteria was shown (29). Clinical evidences showed that vaginal creams made of garlic were effective in improvement of signs and complications of the mixed BV (30).

Exact mechanism of allicin and garlic concentrate antimicrobial effect have not been specified yet; however, several mechanisms are proposed. Cross-linking of proteins and SH enzymes is one of these mechanisms. Allicin irreversibly inhibits SH-protease and NADP (+)-dependent alcohol dehydrogenase. It seems that cells of mammals resist severe effects of allicin. One of the reasons for this resistance is the glutathione inside the mammals’ cells that can neutralize the allicin activities. In other words, this is one of the reasons behind the broad range of allicin antimicrobial effects without any adverse effects on the host cells (31). The present study showed significant improvement in Amsel’s clinical criteria after treatment with garlic and metronidazole. In this respect, garlic was found to be more effective than metronidazole. Hafizi et al. showed that micosin vaginal cream has similar efficacy to metronidazole vaginal gel in improving Amsel’s criteria. Clinical improvement based on Amsel’s criteria was estimated at 80% and 70% in micosin and metronidazole treatment groups, respectively (24). Schwebke and Desmond (32) reported 76.8% improvement in Amsel’s criteria after treatment with metronidazole. In another study, improvement rate of Amsel’s clinical criteria at five days and at one month after treatment with metronidazole was reported as 83.3% and 70%, respectively (33). In the present study, the majority of subjects in garlic and metronidazole groups had white homogenous discharge. In a study, white homogenous discharge was reported in 87% of women with BV (34). Simbar et al. reported 100% prevalence of gray-white homogenous discharge among their study subjects in both metronidazole and oregano groups (35). Whiff test has a sensitivity of 33.9% and a specificity of 86.9% (36). In the present study, the majority of subjects in garlic and metronidazole groups showed positive results in Whiff test. In a study, this test was positive in 94% and 92% of subjects in garlic product and metronidazole groups, respectively (24).

Clue cells have a sensitivity of 76.7% and a specificity of 92.4%; thus, it is the most valuable criterion in diagnosis of BV (37). High specificity of clue cells (86%) in diagnosis of BV was reported in a study (38). The present study showed that the majority of subjects in both groups had clue cells in the wet prep examination. A study reported that 93% of BV patients had clue cells in the wet prep examination (39). Vaginal discharge pH is one of the most sensitive (sensitivity of 97%) Amsel’s criteria, but has a low specificity of 26% (38). In the present study, the majority of subjects in both groups had pH > 4.5. Hafizi et al. reported that 84% of the subject in micosin group and 74% in metronidazole group had pH > 4.5 in their vaginal discharge (24).

In this study, stained smear results of zero to three according to Nugent criteria were considered as mean laboratory improvement. In a study, Nugent criteria sensitivity of 96% and specificity of 99% were reported (40). In the present study, the majority of subjects in both groups had Nugent-based Gram stain results of zero to three after treatment and had the same laboratory improvement. Hafizi et al. demonstrated that the diagnosis of BV was made in 56% and 52% of samples in micosin and metronidazole groups, respectively, through based on Nugent criteria, which is in line with our study (24).

In this study, treatment success rate was considered as improvement in combined clinical and laboratory criteria. There was no significant difference between garlic and metronidazole groups in terms of treatment success rate. Khalifa Soltani demonstrated the efficacy of vaginal creams, which were made of garlic, in treatment of mixed vaginitis is 80% of patients (30). Studies have demonstrated that *Mycoplasma* and *Mobiluncus*, which play an important role in developing BV, are resistant to antibiotics (41). Bradshaw et al. showed that concurrent improvement rates of Amsel’s and Nugent criteria in BV in oral metronidazole group were 85% (42).

In this study, there was a significant difference between the two treatment groups in terms of complications as metronidazole caused more side effects. Verstraelen et al. stated that patients that had used metronidazole had many gastrointestinal side effects such as nausea, vomiting, diarrhea, abdominal cramps, heartburn, and metallic taste in the mouth (11). Treatment of BV with recommended oral or vaginal antibiotics is often associated with treatment failure or high rates of recurrence (43).

### 5.1. Strong Points of Study

Considering high prevalence of this infection, high probability of its recurrence, and due to numerous side effects of metronidazole, use of an herbal alternative with the same effect but fewer side effects is an effective way to resolve this problem.
